# Development and psychometric properties of a friendly dietary function assessment scale for home-dwelling people with dementia

**DOI:** 10.1186/s12912-023-01314-9

**Published:** 2023-05-05

**Authors:** Mei-Yin Liu, Hua-Tsen Hsiao, Yi-Ju Chen, Chi-Jane Wang, Jing-Jy Wang

**Affiliations:** 1 Department of Nursing, College of Medicine, National Cheng Kung University, Tainan City, Taiwan; 2 Department of Nursing, National Tainan Junior College of Nursing, Tainan City, Taiwan; 3 Siaying District Health Station, Public Heath Bureau of Tainan Government, Tainan City, Taiwan

**Keywords:** dietary, function, instrument, dementia

## Abstract

**Background:**

Mealtime difficulties related to cognitive functioning negatively impact a patient’s life during the various stages of dementia, and they typically cause a burden and stress on family caregivers. Most people with dementia live at home alone or are cared for by informal caregivers, typically their spouses or other family members. However, no suitable screening tools for home-dwelling patients with dementia have been developed, nor have measurements focused on executive and self-eating functions. This study aimed to develop and evaluate the psychometric properties of the Dietary Function Assessment Scale (DFAS) for community-dwelling persons with dementia.

**Methods:**

A mixed-method design was used to develop the instrument. Methods included a comprehensive literature review to identify the item pool and an expert panel to assess the initial item pool. We performed convenience sampling of 190 home-dwelling people with dementia for psychometrical evaluation. The psychometric properties tests included item and factor analyses, criterion-related validity testing, internal consistency reliability testing, and defining the optimal cut-off values. The study was conducted from 2018 to 2019.

**Results:**

Items were generated based on an extensive literature review and pre-existing scales related to mealtime and executive functions in persons with dementia. The S-CVI/Ave of the DFAS was 0.89. A Principal Component factor analysis demonstrated seven items, with a two-factor structure accounting for 56.94% of the total variance. The two extracted factors were Self-eating ability and Dietary executive function. The confirmatory factor analysis indicated a good model fit. The criterion-related validity was adequate (r = -0.528, p < 0.01). The reliability of Cronbach’s alpha internal consistency was 0.74, and McDonald’s Omega coefficient was 0.80; the optimal cut-off value of 13 points with an AUC of 0.74 was established to determine poor dietary functioning in persons with dementia.

**Conclusion:**

The DFAS was simple, user-friendly, and a valid and reliable instrument to assess dietary functioning in community-dwelling persons with dementia. This short scale can be helpful for caretakers, who can use it to identify the dietary needs of home-dwelling persons with dementia and improve their care and eating experience.

## Background

Cognitive dysfunctions in memory, thinking, problem-solving, and emotional problems severely interfere with the daily lives of people with dementia [1]. Among them, the dietary function is essential to daily life [2]. People who suffer from earlier stages of dementia may experience executive dysfunction [3]. This primarily affects their planning and decision-making related to nutritional intakes, such as shopping, food preparation, food choices, and intake amount [3–5]. As the disease progress, they may gradually experience eating and feeding problems or other mealtime difficulties including the inability to concentrate on eating, inability to place food into the mouth, food falling from the mouth, failure at spoon-feeding [6, 7], difficulty in chewing and swallowing, or altered eating behaviours during mealtime [5, 8, 9] due to apraxia (inability to use utensils) and agnosia (inability to recognise food) [10].

About half the people with dementia have lost their self-feeding abilities within eight years post-diagnosis [11]. Many studies have found that 38.6% [12] and 36% of people with dementia have eating difficulties or other problems [13] that contribute to the progression of dementia. Eating problems make mealtime slow and result in many adverse health outcomes for people with dementia, including weight loss, aspiration, infection, dehydration, being at risk for malnutrition, and death [5, 9, 14–16]. Dietary and nutritional problems related to cognitive disabilities negatively impact a patient’s life during different stages of dementia and cause caregivers distress.

Most people with dementia live at home alone or are cared for by informal caregivers, typically their spouses or other family members [5, 17]. Therefore, accurate and regular assessment or screening of dietary functioning is recommended to detect nutritional problems as early as possible [5, 6, 8, 14]. Aselage [14] conducted an integrative review of 12 instruments divided into three categories, including eating behaviours (Level of Eating Independence Scale, LEI; Eating Behaviour Scale, EBS), feeding behaviours (Feeding Abilities Assessment, FAA; Edinburgh Feeding Questionnaire, EdFED-Q; Self-feeding Assessment Tool of Osborn and Marshall; McGill Ingestive Skills Assessment, MISA; Feeding Behaviours Inventory, FBI; Feeding Traceline Technique, FTLT; Feeding Dependency Scale; The Aversive Feeding Behaviour Inventory, AFBI) and mealtime behaviours (Meal Assistance Screening tool, MAST; Structured Meal Observation, SMO). However, only three instruments (EdFED-Q, FAA, and FTLT) were psychometrically evaluated. Moreover, AFBI, EdFED-Q, and EBS were used to assess the nutritional status of moderate to severe people with dementia in long-term care facilities. These instruments typically focus on the nutritional situation. They often include questions on BMI, weight loss, reduced dietary intake, and disease stress for people with dementia who live in care facilities. However, people with dementia in institutions do not need to prepare their own meals. In addition, these instruments are not simple or short enough for informal caregivers to administer at home, i.e., none of these tools have been specifically designed and validated for home-dwelling persons with dementia. Furthermore, few suitable screening tools have been applied to executive and self-eating functions in the home-dwelling of people with dementia [5]. Therefore, developing a user-friendly instrument to assess dietary functioning that covers both executive and self-eating functions for home-dwelling people with dementia is necessary. Such an instrument will help family caregivers identify issues with dietary functioning earlier and provide appropriate assistance and interventions to help this population. This study’s purpose was to develop and evaluate the psychometric properties of the Dietary Functional Assessment Scale (DFAS) for use in home-dwelling people with dementia.

## Methods

### Study design

**Fig. 1:**
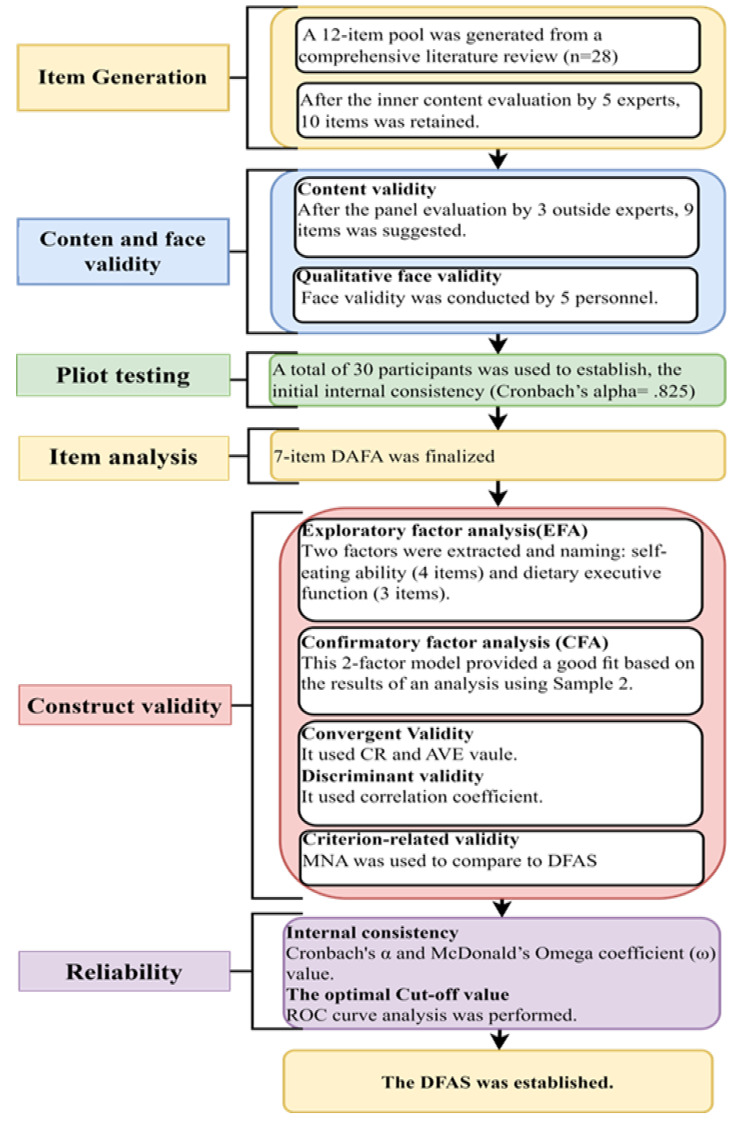
Study flow chart

A mixed-method design was used to develop this study’s instrument, including a comprehensive literature review to identify the item pool and an expert panel to evaluate the initial item pool. We conducted convenience sampling to recruit 190 home-dwelling people with dementia for psychometrical evaluation (see Fig. 1). Instrument development included the following: item generation, content and face validity evaluation, pilot testing, and psychometric analysis [18]. The optimal cut-off value was based on the receiver operator characteristic (ROC) analysis method in terms of scale development.

### Item generation

Items were generated through three stages. Stage (I) Systematic Searching: an extensive literature review based on pre-existing scales related to eating, mealtime behaviours, and executive functions in people with dementia. Stage (II) Item selection: through peer reviews, 11 instruments out of 28 articles were referenced for item generation of DFAS. The initial 12 items were adopted from the instruments mentioned above. Stage III: Item evaluation: five internal research team members who were dementia care experts were asked to evaluate the item pool. Based on the insiders’ comments, one item, “Eat meals at a normal pace and with appropriate manners,“ was removed due to the concept being unclear. Ten items were retained and sent to an outside expert’s panel. Each item on the scale was rated by the panel on a 4-point Likert scale of 0 (no idea), 1 (can do), 2 (seldom), and 3 (cannot do).

### Content and face validity

An expert panel consisting of three professionals with over 10 years of experience in dementia care and research, including a community nutritionist, a home-based occupational therapist, and a dementia care specialist, evaluated the content validity of the DFAS. Each expert individually rated the relevance, applicability, representativeness, and clarity of each of the 10 items using a 4-point Likert-type scale (1 = not relevant to 4 = relevant). The CVI calculation was based on item-level CVI (I-CVI) and an average summary content validity index (S-CVI/Ave) by the total number of experts. According to Polit, Beck [19], an I-CVI of 0.79 or over was appropriate, while an I-CVI of 0.7 to 0.9 and less than 0.7 was revised and eliminated, respectively. Furthermore, S-CVI/Ave values of ≥ 0.9 indicated the relevance, clarity, simplicity and comprehensiveness of the scale [20].

The face validity was examined by inviting five relevant participants, including one home-based care provider, two family caregivers, one nutritionist, and one dementia specialist. The difficulty level, wording, and comprehensiveness of the scale were examined [21].

### Pilot testing

A pilot study was conducted to ensure the scale’s language, wording, item difficulty, understanding, time to complete, and internal consistency. The sample size for pilot testing was recommended to be at least 30 participants [22]. The DFAS was pilot-tested with a group of family caregivers of home-dwelling people with dementia who met the study criteria (described in the sample section) at neurology outpatient clinics. A total of 30 participants were enrolled using convenience sampling, for which Cronbach’s alpha was 0.825.

### Psychometric evaluation of the DFAS

1. ***Sample.*** Through a convenience sampling procedure, family caregivers who met the following criteria and were visiting the participating hospitals’ neurology outpatient clinics were recruited: (1) have been serving as the primary caregiver of the home-dwelling People with Dementia for at least six months. (2) Their care recipients have a diagnosis of dementia or Mini-Mental State Examination (MMSE) score < 26 (graduated from senior high school) and < 18 (under senior high school) or a Clinical Dementia Rating (CDR) ≥ 1 and have food preparation or eating difficulties during mealtimes. People with dementia who underwent artificial feeding (tube feeding) and living in care facilities were excluded. Based on the ratio of 5–10 participants to one item [18], a minimum sample size of 190 participants was targeted.
2. ***Data Collection Procedure.*** The study was approved by the ethical committee of National Cheng Kung University (No. IRB# ER 103–414). Data were collected anonymously. Each eligible patient participant had written proxy consent from their family caregiver or guardian if they were unable to consent. Demographic information, which included each patient’s age, gender, education, work, marital status, years of morbidity, MMSE score, CDR, current eating ability, and MNA score, was collected. This was followed by administering the DFAS. Family caregivers were asked to collect the data via pen and paper.
3. ***Instrument.*** The Chinese version of the Mini Nutritional Assessment (MNA) was used to establish criterion-related validity. The MNA is widely used for assessing nutrition status, including malnutrition or risk of malnutrition, and has been reported as having adequate psychometric properties [23]. The MNA consists of 18 self-reported questions. A score of ≤ 23.5 indicates a risk for malnutrition and ≤ 17 for malnutrition where higher scores indicate that the person is well-nourished [24]. The internal consistency Cronbach’s coefficient alpha for the DFAS was greater than 0.7. The sensitivities of the MNA ranged from 81 to 100%, and the specificities ranged from 36 to 98% [25].
4. ***Data Analysis.*** Data were analysed using the SPSS v. 17 (SPSS Inc., Chicago, IL, USA) and the AMOS v. 20 (IBM Corp., NY, USA) statistical software packages. Descriptive statistics, including means, standard deviations (SDs), frequencies, and percentages, were performed. Construct validity included an exploratory factor analysis (EFA) and a confirmatory factor analysis (CFA); therefore, the participants were randomly split into Samples 1 and 2. Chi-square and independent t-tests were used to establish the differences between the two groups. Group 1 was used for the item analysis and the EFA. The factor was extracted using a principal component analysis (PCA) with a varimax rotation. The Kaiser-Meyer-Olkin (KMO) was used to measure sampling adequacy (> 0.6); Bartlett’s Test of Sphericity (*p* < 0.001) indicated sampling adequacy of the data set; a screen plot was used to determine the factor number. Furthermore, a CFA was conducted on the data from Group 2 based on the EFA results. We set a factorial loading of 0.30 as the minimum coefficient necessary for the factor analysis [26]. We hypothesised that relationships between the DFAS and the MNA would be negative for the criterion-related validity, where a Pearson’s correlation coefficient of at least 0.3 was required [27]. Cronbach’s alpha test and McDonald’s Omega coefficient were used to determine the internal consistency reliability; both were higher than 0.7 and were considered reliable [28, 29]. The optimal cut-off values were based on the receiver operator characteristic (ROC) curve and the area under the curve (AUC) [30].

## Results

### Demographic characteristics

A total of 190 family caregivers of people with dementia participated in the study. 66.3% of the caregivers were female, and the mean age was 56.3 years (SD = 12.81). Almost three-quarters (74%) had completed high school. The care recipients’ average age was 77.39 years (SD = 9.10), and most were female (59.5%). Most of the subjects had mild to moderate dementia, and over half of the participants (53.7%) were at risk malnutrition (Table 1).

**Table 1:** Demographics of the Participants and Between-Group Differences

Variables	All (n = 190)	Sample 1 (n = 95)	Sample 2 (n = 95)	${x}^{2}$/ *p* value
Gender				0.301
Male _n (%)	77 (40.5%)	35 (36.8%)	42 (44.2%)	
Female _n (%)	113 (59.5%)	60 (63.2%)	53 (55.8%)	
Age _ mean (SD)	77.39 (9.10)	77.65 (10.16)	77.13 (7.49)	0.691
Education				0.908
Illiterate _n (%)	38 (20.0%)	20 (21.0%)	18 (18.9%)	
Lower junior high school _n (%)	106 (55.8%)	53 (55.8%)	53 (55.8%)	
Upper junior high school _n (%)	46 (24.2%)	22 (23.2%)	24 (25.3%)	
Marital status				0.230
Marriage (partner) _n (%)	123 (64.7%)	54 (56.6%)	69 (72.6)	
Single _n (%)	67 (35.3%)	41 (43.2%)	26 (27.4%)	
Morbidity years _ mean (SD)	7.17 (3.07)	7.20 (3.17)	7.14 (2.98)	0.882
MMSE (n = 170) _ mean (SD)	14.51 (5.68)	14.28 (5.65)	14.75 (5.68)	0.590
CDR (n = 51)				0.428
0.5 _n (%)	2 (3.9%)	0 (0%)	2 (7.7%)	
1 _n (%)	16 (31.4%)	8 (30.2%)	8 (30.8%)	
2 _n (%)	23 (45.1%)	13 (52.0%)	10 (38.5%)	
3 _n (%)	10 (19.6%)	4 (16.0%)	6 (23.1%)	
Current eating ability				0.037
Independent _n (%)	133 (70%)	60 (63.2%)	73 (76.8%)	
Assistance _n (%)	45 (23.7%)	30 (31.6%)	15 (15.8%)	
Feed _n (%)	12 (6.3%)	5 (5.3%)	7 (7.4%)	
Food preference change				0.557
No _n (%)	110 (57.9%)	53 (55.8%)	57 (60.0%)	
Yes _n (%)	80 (42.1%)	42 (44.2%)	38 (40.0%)	
MNA score (n = 188)				0.314
Normal (>24) _n (%)	44 (23.4%)	20 (21.1%)	24 (25.8%)	
At risk (17-23.5) _n (%)	101 (53.7%)	49 (51.6%)	52 (55.9%)	
Malnutrition (<17) _n (%)	43 (22.9%)	26 (27.4%)	17 (18.3%)	

*Notes.* SD, standard deviation; MMSE, Mini-Mental State Examination; CDR, Clinical Dementia Rating. ^*^p value < 0.05

### Validity

#### Content and face validity

Through an internal expert evaluation, the initial I-CVI ranged from 0.67 to 1.00 for the 10 initial items. Therefore, we deleted two inappropriate items, and one item was added based on the comments of the experts. As such, the modified version of the DFAS comprised nine items. Three external experts were invited to evaluate the content validity in two rounds. Consequently, the S-CVI was 0.9. Each item was rated by the respondent on a 4-point Likert-type scale ranging from 0 to 3, with the total score ranging between 0 and 27, where higher scores reflected more functional limitations. Face validity was conducted, and the wording was revised based on comments.

#### Item analysis

Table 2 presents the item analyses for the nine-item DFAS. The mean, SD, comparisons of extreme groups, and item-overall score correlation (*r*) were used in the item analysis for item selection. Considering the results of the overall analysis for the average, standard deviation, composite reliability (CR) value, and correlation of all items, Item 6 was deleted due to a low SD, a non-significant CR value, and a low item-total correlation (*r* = 0.253) below 0.30. Item 9 was deleted due to a large mean (mean = 2.66 > 2.58). Therefore, a seven-item DFAS was established at this stage.

**Table 2:** Item Analysis of the 9-Item Diet Function Assessment Scale

Items		Mean (SD)	t (CR value)	Corrected Item-Total Correlation
1.	Decides that they need to eat	1.71 (0.99)	-8.59^***^	0.60
2.	Chooses appropriate utensils when eating	1.46 (0.77)	-7.83^***^	0.70
3.	Eats an appropriate amount of food	1.91 (0.92)	-4.92^***^	0.49
4.	Uses a spoon to eat	1.57 (0.90)	-8.60^***^	0.58
5.	Needs to have foods cut into small pieces	1.73 (0.94)	-7.45^***^	0.53
6.	Only uses fingers to eat food	1.08 (0.40)	-1.79	0.25
7.	Relies on others to be fed	1.34 (0.72)	-5.11^***^	0.65
8.	Decides to prepare a light meal or snack for self	2.57 (0.81)	-18.62^***^	0.62
9.	Prepares or cooks a light meal or snack safely	2.66 (0.74)	-7.18^***^	0.52
	Overall Mean ± 1.5 SD	1.78 (0.53) (2.58, 0.99)		

*Notes.* N = 95 (Sample 1). *p value < 0.05, **p value < 0.01, ***p value < 0.0001

#### Exploratory factor analysis (EFA)

The EFA using a principal component analysis (PCA) with a varimax rotation was performed for Sample 1. The Kaiser-Meyer-Olkin value for the eight items was 0.734, and Bartlett’s test of sphericity value showed statistical significance (${x}^{2}$ = 279.530, *df* = 28, *p* < 0.000). This indicates that the data were suitable for the factor analysis. Seven items were analysed through an EFA. Two factors were extracted based on the screen plot; eigenvalues greater than 1.0 indicated an appropriate factor structure (Table 3). Factor 1: *self-eating ability* included four items (1, 2, 3, and 8); Factor 2: *dietary executive function* included three items (5, 4, and 7). The cumulative explanatory power of the second-factor analysis was 56.94%.

**Table 3:** Exploratory Factor Analyses Results

Factor/ Item	Factor loading		Cumulative Variance (%)	Cronbach’s alpha	McDonald’s Omega coefficient
	1	2			
Factor 1: Self-eating ability			42.96	0.70	0.70
1. Decides that they need to eat	**0.77**	0.12			
3. Eats an appropriate amount at meals	**0.68**	0.11			
2. Chooses appropriate utensils when eating	**0.67**	0.45			
8. Decides to prepare a light meal or snack for themself	**0.63**	0.19			
Factor 2: Dietary executive function			13.98	0.69	0.73
4. Uses a spoon to eat	0.16	**0.79**			
5. Need to have food cut up into small pieces	0.10	**0.77**			
7. Relies on others to be fed	0.34	**0.73**			
Total Variance Explained /Cronbach’s alpha/ McDonald’s Omega			56.94	0.74	0.80

*Note.* N = 95 (Sample 1). The number in bold indicates the factor loading of including in Diet Function Assessment Scale items

#### Confirmatory factor analysis (CFA)

**Fig. 2:**
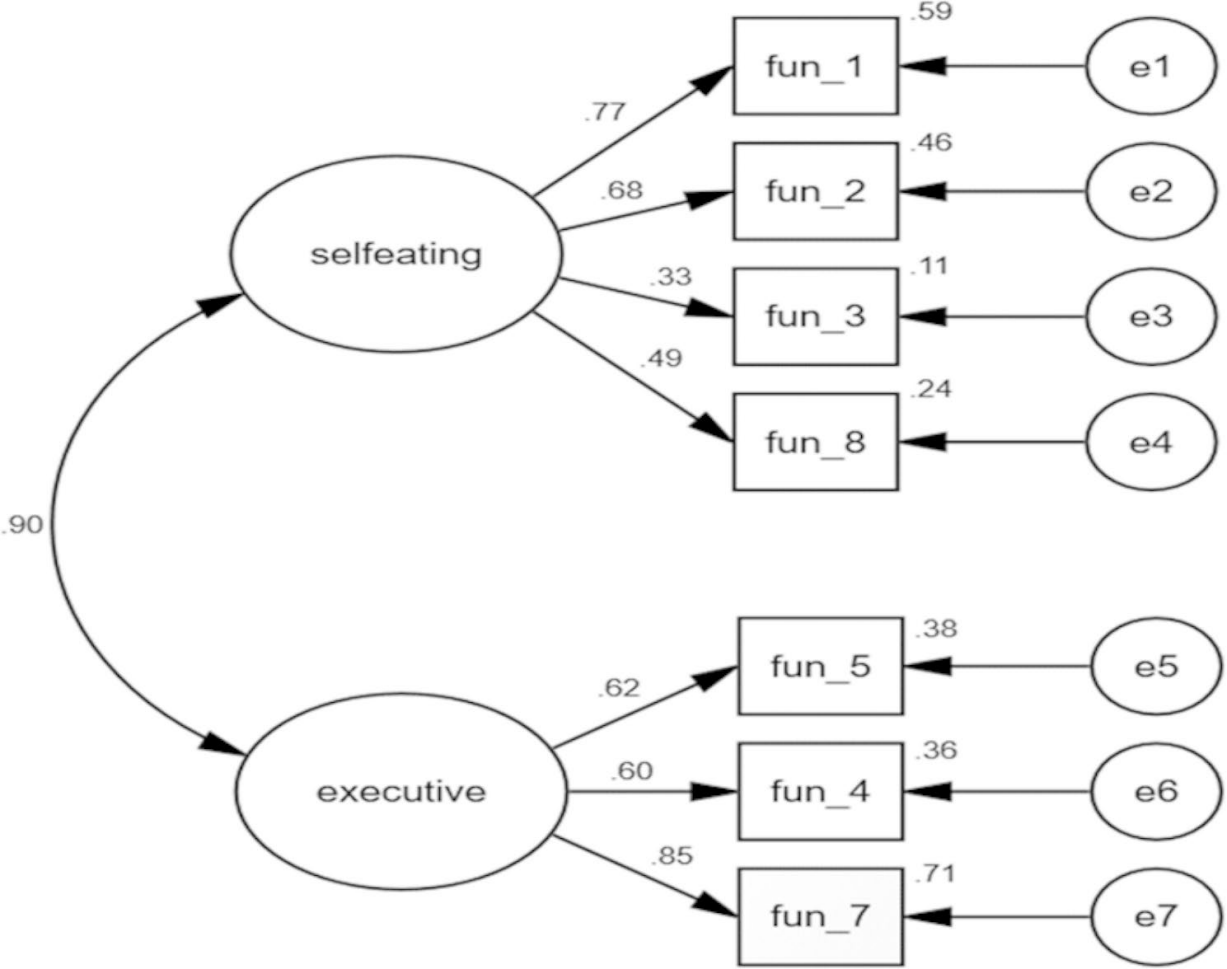
**Confirmatory Factor Analysis of the 7-item Dietary Function Assessment Scale (Sample 2, n = 95)** Model fit: Chi-square (χ2) = 17.51; degrees of freedom (df) = 13; χ2/df (CMIN/DF) = 1.35 (p = 0.18); Standardised root mean square residual (SRMR) = 0.05; Root mean square error of approximation (RMSEA) = 0.06; Tucker-Lewis Index (TLI) = 0.96; comparative fit index (CFI) = 0.97.

This study’s 2-factor model provided a good fit based on the results of an analysis using Sample 2 (Fig. 2). The results indicated a Chi-square (${x}^{2}$) = 17.51; degrees of freedom (*df*) = 13; ${x}^{2}$ / df (CMIN/DF) = 1.35 (*p* = 0.18); standardised root mean square residual (SRMR) = 0.05; a root mean square error of approximation (RMSEA) = 0.06; Tucker-Lewis index (TLI) = 0.96 and a comparative fit index (CFI) = 0.97. Table 4 presents the standardised factor loadings (SFLs), standardised error, and the squared multiple correlations (R^2^) for the 7-item DFAS. Most of the SFLs were over 0.50 (ranging from 0.33 to 0.85), except for item 3 (SLF = 0.33), which was a reliable item for measuring self-eating ability, and we, therefore, retained it. The CFA results supported the factor structure of the 7-item DFAS.

**Table 4:** SFL, R^2^, and SE of the DFAS

Dimension		Items	SFL(t)	SE	R^2^
Self-eating ability	1	Decides that they need to eat	0.77(7.84)	0.38	0.59
	3	Eats an appropriate amount at meals	0.33(2.95)	0.43	0.11
	2	Chooses appropriate utensils when eating	0.68(6.72)	0.38	0.46
	8	Decides to prepare a light meal or snack for self	0.50(6.07)	0.43	0.24
Dietary executive function	4	Uses a spoon to eat	0.60(5.85)	0.41	0.36
	5	Need to have foods cut into small pieces	0.62(6.07)	0.43	0.38
	7	Relies on others to be fed	0.85(8.92)	0.33	0.71

*Notes.* N = 95 (Sample 2). SFL = standardised factor loadings; SE = standardised error; R^2^ = squared multiple correlation

Table 5 presents the convergent validity of the two sub-scales, Self-eating ability and Dietary executive function. The composite reliability (CR) of the two sub-scales achieved 0.70, and the average variance extracted (AVE) achieved 0.40. This indicates that the indicators of the two sub-scales merged to determine self-eating ability and dietary executive function, respectively.

Regarding discriminant validity, the results indicate that the correlation coefficient of the two sub-scales (0.59) was lower than the square root of AVE /DV (0.70 and 0.73). This suggests that the two latent variables deviated from each other [31].

**Table 5:** Convergent and Discriminant validity of the DFAS

Dimension	CR	AVE	Self-eating ability	Dietary executive function
Self-eating ability	0.70	0.40	(0.63)	
Dietary executive function	0.74	0.50	0.59	(0.70)

Notes. CR = composite reliability; AVE = average variance extracted; ( ) = the square root of AVE.

#### Criterion-related validity

The results showed a significant negative correlation between the DFAS and MNA scores (r = -0.528, p < 0.01).

### Reliability

#### Internal consistency

Cronbach’s α and McDonald’s Omega coefficient (ω) were used to examine the seven items’ internal consistency. Cronbach’s α in each factor ranged between 0.69 and 0.70, and the overall Cronbach’s α value was 0.74; McDonald’s Omega coefficient in each factor ranged from 0.70 to 0.73, and the overall McDonald’s Omega coefficient value was 0.80.

#### The optimal cut-off value

To evaluate the optimal cut-off value of the DFAS score to detect risks related to dietary functioning in people with dementia, a ROC curve analysis was performed using an MNA cut-off value of ≤ 23.5 (at risk for malnutrition) and ≤ 17 (malnutrition) as the gold standard. The optimal cut-off value was selected using the criterion based on Youden’s Index (Hajian-Tilaki, 2018) and defined as YI = maxc Se (c) + Sp (c) − 1, which maximises the sensitivity and specificity of the results. The AUC was 0.51, and the optimal cut-off score to determine risks related to dietary functions was 11. The cutoff yielded sensitivity/specificity values of 0.87/0.21. The AUC was 0.74, and the optimal cut-off score to determine poor dietary functioning was 13, with the cutoff yielding sensitivity/specificity values of 1.00/0.53.

## Discussion

This study’s newly developed DFAS is primarily used by family caregivers to examine the dietary functioning of home-dwelling people with dementia. This study’s findings verify that the DFAS is a user-friendly instrument with good validity and reliability. The results suggest that this tool is a reliable means by which dietary functions among home-dwelling people with dementia can be measured. A 10-item scale was initially generated through a literature review and based on the clinical experience of the research team. After evaluation and recommendations from experts, two unsuitable items were deleted, and one item was added, where the final scale comprised nine items. Acceptable content validity of greater than 0.80, according to Waltz, and Strickland [20], was obtained. Through an item analysis and EFA, two additional items were deleted that could not meet the criteria. Factors that emerged in these EFA appeared relatively stable, well-defined, and conceptually coherent, with two factors explaining 56.94% of the variance. Thus, the final DFAS includes seven items and two factors: self-eating *ability* and *dietary executive function.* The CFA analysis conducted using Sample 2 also indicated that the scale category goodness-of-fit indices were appropriate based on Hays and Revicki’s [32] recommendation that reliabilities exceeding 0.70 are considered acceptable.

The first factor, *self-eating ability*, refers to the ability to eat. This involves essential dietary-related functions in daily life, such as eating habits, putting food into the mouth, using utensils, healthy food preferences, and adequate dietary intake during the eating process. Maintaining a patient’s eating ability is a significant concern for family caregivers. Among the items related to habits and food preferences influenced by cognitive decline corresponding to the results of other studies [10], changes in the eating behaviours of people with dementia lead family caregivers to be concerned about their emotional status. Although in the CFA, item 3 (eats an appropriate amount at meals), there was a slightly lower factor loading on this factor, it was still higher than 0.3. Some eating behaviour assessment tools used for people with dementia consider intake amount to be directly associated with functions and nutritional status. Thus, item 3 was retained [6, 14].

The second factor, *dietary executive functions*, is an essential aspect of dietary functioning, which indicates planning, meal preparation, and making decisions at mealtime. Impairments in executive functions can affect the performance of instrumental daily life activities and worsen the quality of life [33]. Executive functions have already been indicated to deteriorate prematurely in people with dementia, even starting from mild cognitive impairment [34]; this may be the earliest symptom that accompanies cognitive decline, further affecting the ability to prepare meals and eat [35, 36]. Patients in advanced stages of dementia are often institutionalised due to increasing dependence. Therefore, most previously developed assessment instruments have been concerned about care needs, such as assisting with feeding, feeding behaviours, choking, swallowing impairment during mealtime, and nutritional status. It would be worthwhile to examine the impairment in executive functions of home-dwelling people in the early stages of dementia.

This study’s findings indicate that the total DFAS score had a moderate negative correlation (*r* = -0.528, *p* < 0.01) with the MNA score, demonstrating that poor dietary functioning is associated with poor nutritional status. This result is similar to the findings of previous studies on care facilities that have found impairments in “eating ability” and “meal preparation” were significantly related to malnutrition [37–40].

The present study proposes the AUC approach to identify the optimal cut-off point value through ROC analysis. The results showed poor AUC and specificity values for the cut-off score of 11 points. However, the results indicate satisfactory AUC and sensitivity/specificity values for a cut-off score of 13 points. Therefore, we recommend this cut-off point to detect poor dietary functioning in home-dwelling people with dementia.

Measurements developed to assess concepts that are different but similar to the concept of mealtime, feeding, and eating of people with dementia have been developed and used to measure mealtime difficulties since the 1990s [14]. However, most prior measurements have addressed moderately to advanced stages of dementia residing in long-term care facilities and hospital settings. Furthermore, most tools lack good psychometric properties. Among them, only the EdFED-Q [7] is a validated tool [37], yet, the EdFED-Q is an observational tool for use in moderate to advanced stages of dementia in long-term care facilities, not a screening tool for early detection for home-dwelling people with dementia.

We acknowledge this study’s following limitations. First, the small size and convenience sampling nature of the sample. As such, the findings might not reflect the general population, which can be a potential limitation of this study. Secondly, we did not provide construal-related measurements to measure the same target. Last, some psychometric properties are missing such as the test-retest reliability. In addition to improve the psychometric properties of the tool through the COSMIN indicators in future research, the developed tool can be extensively used in communities for early detection of nutritional problems for people with dementia.

## Conclusion

The DFAS is a reliable and valid short scale for assessing *self-eating ability* and *dietary executive function* among home-dwelling people with dementia. This short assessment scale is user-friendly for use in both formal and informal care situations in home settings for the early detection of diet functioning associated with nutritional problems to improve the quality of dietary care. Such a measurement model can be used for future longitudinal studies and intervention evaluations and to determine dietary functioning among people with dementia in the community.

## Data Availability

Availability of data and materials are available upon request from the corresponding author.

## Abbreviations

DFAS: Dietary Function Assessment Scale
I-CVI: Item-level Content Validity Index
S-CVI: Scale-level Content Validity Index
ROC: Receiver Operating Characteristic
AUC: Area Under Curve
MMSE: Mini-Mental State Examination
CDR: Clinical Dementia Rating
LEI: Level of Eating Independence Scale
EBS: Eating Behaviour Scale
FAA: Feeding Abilities Assessment
EdFED-Q: Edinburgh Feeding Questionnaire
MISA: McGill Ingestive Skills Assessment
FBI: Feeding Behaviours Inventory
FTLT: Feeding Traceline Technique
AFBI: The Aversive Feeding Behaviour Inventory
MAST: Meal Assistance Screening tool
SMO: Structured Meal Observation
